# Separating Subjective from Objective Food Value in the Human Insula: An Exploratory Study Using Intracranial EEG

**DOI:** 10.3390/brainsci15060593

**Published:** 2025-05-31

**Authors:** Benjamin Hébert-Seropian, Olivier Boucher, Daphné Citherlet, Manon Robert, François Richer, Dang Khoa Nguyen

**Affiliations:** 1 Département de Psychologie, Université du Québec à Montréal (UQÀM), Montréal, QC H2X 3P2, Canada; 2 Centre de Recherche du Centre Hospitalier de l’Université de Montréal (CRCHUM), Montréal, QC H2X 0A9, Canada; 3 Département de Psychologie, Université de Montréal, Montréal, QC H3C 3J7, Canada; 4 Centre Hospitalier de l’Université de Montréal (CHUM), Montréal, QC H2X 0C1, Canada; 5 Département de Neurosciences, Université de Montréal, Montréal, QC H3T 1J4, Canada

**Keywords:** insula, intracranial EEG, ERP, food images, food cue reactivity, homeostasis, hunger, satiety, EPIC model, alliesthesia

## Abstract

**Background/Objectives**: The human insula is a key structure implicated in integrating internal states and external food cues, yet its precise role remains unclear, in part due to the temporal limitations of neuroimaging techniques like fMRI. To address this gap, we conducted an exploratory study using an intracranial EEG (iEEG) to investigate how the insula encodes both the subjective and objective properties of food-related stimuli, and how this encoding is modulated by hunger and satiety. **Methods**: Eight patients with drug-resistant epilepsy undergoing a pre-surgical evaluation between 2017 and 2023 participated in this study. Depth electrodes implanted in the insular cortex recorded event-related potentials (ERPs) in response to visual food cues. The sessions were conducted in two prandial states (hungry and satiated). The subjective ratings (appetite and palatability) and objective nutritional values (e.g., calories, carbohydrates) were collected and analyzed using paired t-tests, MANOVAs, and partial correlations. **Results**: Hunger increased the ERP amplitudes within the 350–450 ms interval, consistent with the EPIC model and positive alliesthesia, while satiety unexpectedly enhanced the early responses (150–250 ms). Importantly, the neural activity related to nutritional values was largely uncorrelated with the subjective ratings, suggestive of distinct processing streams. The mid- and posterior insula showed greater sensitivity to both subjective and nutritional information than the anterior insula. **Conclusions**: These findings offer novel electrophysiological insights into how the insula differentiates between implicit and explicit food-related signals, depending on the homeostatic state. This work supports a dual-route model of food cue processing, and may inform interventions targeting insular activity in disordered eating.

## 1. Introduction

The insula is a brain region located deep within the Sylvian fissure, obscured by the frontal, parietal, and temporal lobes [1,2]. Often referred to as the “hidden fifth lobe”, research has revealed the central role of the insula in integrating sensorimotor, emotional, and high-level cognitive processes. It is also considered the brain’s centre for interoception—the process by which the nervous system senses, interprets, and integrates signals originating from within the body (for a review, consult Uddin et al., 2017 [3]). Anatomically, it comprises anterior and posterior sections, delineated by the central insular sulcus, with each part exhibiting distinct functions and bidirectional connections to other brain regions [4,5].

Early stimulation studies in humans and primates identified the insula as a key gustatory region, which was later confirmed by neuroimaging and electrophysiological findings [6]. In humans, although some controversy persists regarding the pinpoint location of the gustatory cortex, most research using fMRI paradigms suggests an analogous role in the most caudal aspects of the anterior insula, extending into the mid-insula [7]. Both the anterior and mid-insula respond to a wide array of flavours and are implicated in processing somatosensory stimuli, such as texture, temperature, and capsaicin [8,9,10,11,12].

The anterior insula has been shown to respond to the identity and intensity of taste sensations, independently of hedonic (pleasure-related) or homeostatic (hunger-related) modulation [8]. However, an expanding body of research has highlighted the mid-insula’s involvement in the processing of external food cues, as well as in adapting to fluctuations in internal hunger and satiety signals. Notably, a meta-analysis of human fMRI studies identified the mid-insula, along with the orbitofrontal cortex and the occipital complex, as key areas exhibiting sensitivity to external food cues [13]. Furthermore, research by Simmons et al. [14] found that, while both the anterior and dorsal mid-insula reacted to taste stimuli and were significantly activated by food cues, only the mid-insula’s activity showed a correlation with peripheral glucose levels, suggestive of a distinct role in maintaining homeostatic balance. Anatomically, the mid-insula is ideally positioned to integrate gustatory and interoceptive signals for the purpose of homeostatic balance, as it constitutes a hybrid zone where somatosensory, gustatory, and interoceptive information shares neuronal real estate and likely involves multimodal neurons responding to both types of inputs [15,16].

The Embodied Predictive Interoception Coding (EPIC) model proposes that the mid-insula supports homeostatic regulation by comparing predicted and actual internal states, generating prediction errors when mismatches occur [17,18,19]. Thus, in a state of hunger, the theory is that the discrepancy between the ideal and actual state of the body is significant, generating a strong prediction error. Based on the structural model of information flow elucidated by Barbas and Rempel-Clower [20], the EPIC model suggests that the predominantly granular cortex of the mid-insula is ideally structured for blending ascendant sensory information with anticipatory signals stemming from the less differentiated, agranular anterior insula. The mid-insula is tasked with detecting and relaying back prediction errors to the agranular cortex to adjust predictions. In the context of hunger, one way to reduce the prediction error would be to enhance the motivational salience of food stimuli, a concept often encapsulated as *positive alliesthesia* [21]. While empirical support for the EPIC model is still emerging, fMRI studies have shown that the mid-insula reflects the current homeostatic status [14,22], whereas the anterior insula may signal anticipated changes in internal states [23,24,25,26], consistent with a role in generating predictions.

Traditional models of overeating have emphasized hedonic control, suggesting that the sensory appeal of appetitive foods can override the physiological signals meant to limit intake [27,28]. However, this influence appears short-lived: while palatability may initially guide food choices, its effect diminishes quickly, and studies have shown weak correlations between food preferences and actual consumption in both animals [29,30] and humans [31,32]. Furthermore, the evidence for a dysregulated hedonic system in obesity is limited [33]. An alternative paradigm highlights the role of post-ingestive signals—particularly those processed in the gut—as key drivers of food preference. This is supported by flavour–nutrient conditioning studies, where animals and humans have been shown to develop preferences for flavours paired with caloric content, even in the absence of enhanced taste [34,35]. These findings suggest that learned associations between taste and nutritional value shape consumption more strongly than conscious pleasure alone [27]. According to de Araujo et al. (2020) [27], the insula may mediate this integration by combining unconscious visceral signals (the “low road”) with sensory information from taste, smell, and texture (the “high road”), assigning a reward value to food cues based on internal physiological needs rather than hedonic appeal.

While the last few decades of fMRI research have firmly established the insula as a key region in food cue processing, our understanding remains limited by the slow temporal resolution of hemodynamic signals. This has left critical gaps in the characterization of the rapid neuronal dynamics within distinct insular subregions, particularly in response to the subjective experiences (e.g., appetite, palatability) versus objective nutritional properties (e.g., calorie, fat content) of food cues. Moreover, the impact of changing homeostatic states—such as hunger and satiety—on these neural responses remains poorly defined. To address these gaps, this study leverages the high-temporal-resolution iEEG to precisely examine the timing and amplitude of insular activity during food cue perception. By linking neuronal responses to both the subjective and nutritional attributes of food, and comparing them across prandial states, this work provides novel insight into how the insula integrates explicit and implicit information in the pre-ingestive phase of food evaluation. Because of the insula’s deep anatomical location and proximity to seizure foci, the iEEG recordings of patients undergoing pre-surgical evaluation for epilepsy [36] offer a rare opportunity to investigate these questions with both high temporal and spatial resolution [37].

Building on this rationale, this study was designed to explore the insula’s function in processing external cues related to food, utilizing event-related potentials (ERPs) derived from iEEG recordings of patients with epilepsy. Our main goal was to examine the neuronal response of the insula to food images, and to study how prandial states affect this response. A secondary aim was to investigate the differential activation of the insula in relation to subjective, conscious experiences, and objective, implicitly detected nutritional information. Based on the EPIC model’s premises as well as the evidence stemming from fMRI studies, we anticipated that the insula would exhibit a higher magnitude of activation in response to food images during a state of hunger compared to satiety. Additionally, we predicted that the response to food images would be greater in the mid-insula than in the anterior or posterior insula. We further hypothesized that the neural responses related to the objective nutritional value of food (e.g., fat, carbohydrate, calorie content) would be uncorrelated with the subjective ratings (e.g., appetite, palatability), indicating the distinct processing of implicit and explicit food-related information. Finally, we anticipated that the mid- and posterior insula would show stronger associations with objective nutritional variables compared to the anterior insula, which was expected to exhibit a comparatively weaker activation in response to both objective and subjective food-related properties.

## 2. Materials and Methods

### 2.1. Participants

This study involved 8 patients (3 women, 5 men; 6 right-handed, 2 left-handed) with an average age of 39.5 years (range: 22–58 years) who were undergoing invasive EEG monitoring as part of a pre-surgical evaluation of their drug-resistant seizures. They were recruited between 2017 and 2023. The experiments were conducted in their hospital room at least three days following electrode implantation. Key inclusion criteria required that at least one electrode was positioned in the insular cortex. Exclusion criteria comprised the following: age limits (18–65 years), severe psychiatric disorders or cognitive impairments impacting task performance, and the presence of frequent interictal epileptiform discharges (IEDs) affecting most (>60%) of the signals of interest. A total of 102 electrode contact points were placed across 10 insulae (7 right, 3 left). Participant demographics and surgery-related details are provided in Table 1. Our research hypotheses and analysis plans were established in advance, as detailed in Section 2.4, with additional data-driven analyses clearly identified as such.

### 2.2. Insular Response to and Subjective Rating of Visual Food Stimuli

Prior to this study, participants provided informed consent and completed a brief questionnaire to identify dietary restrictions and any changes in eating experiences since hospital admission. No significant changes or dietary restrictions were reported.

To investigate the insula’s role in food-related processing, participants passively viewed a set of standardized food images. The stimuli, selected for their relevance to North American diets, were sourced from an image database [38]. Amongst the available variables, object size, brightness, contrast, normalized complexity, and spatial frequencies were used as controls for image characteristics. Nutritional values selected for analysis included fat and carbohydrate content per 100 g and in total, as well as the total calorie content. Each food image was displayed for 2 s, immediately followed by two questions presented on bipolar 5-point Likert scales to assess subjective experiences. The first question asked, “To what degree was this image of food appetizing to you?” (1—very disgusting; 2—disgusting; 3—moderately; 4—appetizing; 5—very appetizing). The second question asked, “To what degree did the image of food make you hungry?” (1—not at all; 2—a little; 3—moderately; 4—a lot; 5—substantially). Participants had as much time as needed to respond after each food image, followed by a 2–3 s randomized inter-stimulus interval marked by a white fixation cross.

This study was conducted in two sessions on one morning. The first session took place after a 10 h fast (hungry condition), while the second occurred 15 min post-breakfast (satiated condition). Although the exact nutritional value of the breakfast was not controlled for, participants were advised to eat until full. Sessions lasted between 30 and 45 min, depending on response times, featuring 70 non-food and 90 food stimuli per session (a total of 140 non-food and 180 food images). The food images represented various categories, including bread, cheese, beverages, full meals, vegetables, fast food, fruit, and sugary treats.

For data collection, a computer with Presentation software (Version 17.0, Neurobehavioral Systems, Inc., Berkeley, CA, USA) was used, positioned approximately 80 cm from the participants at eye level. Participants were seated in their hospital bed, entering their responses using a keyboard on a wheeled tray. To ensure minimal distraction, the room’s door remained closed, and the experiment was monitored via the epilepsy service’s camera system.

### 2.3. iEEG Recording and Analyses

EEG data were captured using a Stellate Harmonie audio–video–EEG monitoring system (Natus Medical, San Carlos, CA, USA) for the first participant, and via a Nihon Kohden EEG system (Nihon Kohden America, Irvine, CA, USA) for all subsequent participants. All EEG data were sampled at a rate of 2 kHz. The recording equipment included deep electrodes, subdural strips, and/or electrode grids provided by Ad-tech Medical Instruments (Racine, WI, USA). Typically, each patient’s EEG recording involved over a hundred contacts, with around ten of these contacts situated within the insula. Insular depth electrode contacts were 1.1 mm in diameter and 2.3 mm in length, spaced at intervals of 5 mm from centre to centre. A mastoid electrode served as the reference, and another as the ground. Exact localization of each electrode’s contacts was verified using post-implantation magnetic resonance imaging. Each insular electrode’s position was categorized based on its caudo-rostral (anterior, middle, and posterior) location. The mid-insula was specifically defined as the narrow region adjoining each side of the central insular sulcus, effectively covering half of the short posterior insular gyrus and half of the long anterior insular gyrus, vertically. Due to the limited sample size, dorso-ventral localization was not considered for analysis.

The EEG data underwent pre-processing and cleaning using Brain Vision Analyzer. High- and low-pass filters were applied at 0.1 and 30 Hz, along with a 60 Hz notch filter. Stimulus-onset markers were automatically detected and inserted, followed by manual inspection for accuracy. Non-insular channels were initially retained only to assist in identifying IEDs, and were later removed. Each participant’s insular EEG data were re-referenced against the average of all insular electrode contacts, excluding any with significant artifacts. Automatic artifact rejection, complemented by manual inspection, was employed to eliminate IEDs and noise-containing epochs. Out of 102 insular channels, 28 were excluded from further processing and analysis due to excessive noise, leaving 74 contact points. Three cases were excluded from this study due to excessive IEDs. In the remaining participants (N = 8), trial retention rates were comparable across conditions and individual cases, averaging 58% (range 40–77%). To enhance signal clarity and spatial localization, bipolar montages were created for each participant’s insular electrodes, followed by offline downsampling to 500 Hz, resulting in 60 bipolar pairs of electrodes.

The processed data were exported to EEGLAB 2022.0 and ERPLAB 9.0, open-access Matlab plugins from the Swartz Center for Computational Neuroscience (Delorme & Makeig, 2004; Lopez-Calderon & Luck, 2014, [39,40]). EEG signals were segmented from −200 to 800 ms post-stimulus for each task condition (food vs. non-food; hungry vs. satiated), with baseline correction applied at −200 ms pre-stimulus.

**Figure 1:**
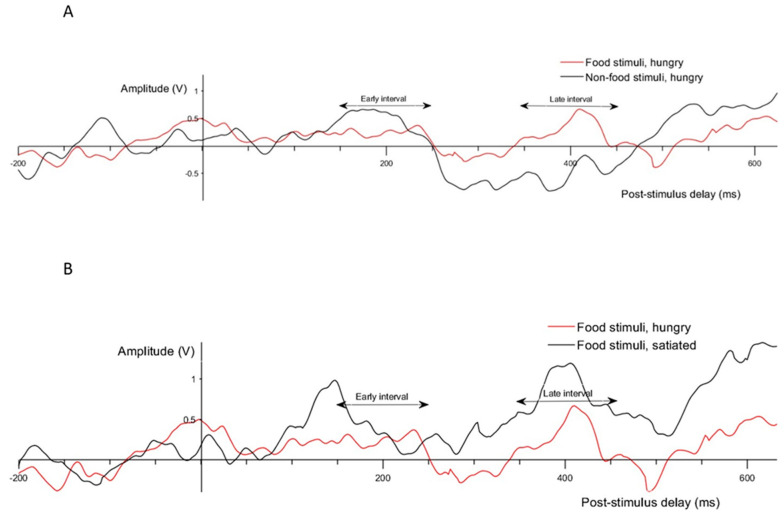
Grand averages of epochs comparing (**A**) food against non-food stimuli in the hungry condition, and (**B**) response to food images in hungry against satiated conditions.

Due to the absence of prior intracranial EEG studies on food cue processing in the insula, the selection of ERP intervals was guided by a combination of data-driven observations and theoretical considerations. Visual inspection of grand-average waveforms comparing food versus non-food stimuli in the hungry state, and food stimuli across prandial states (Figure 1), identified two intervals of interest: 150–250 ms and 350–450 ms. These are consistent with prior ERP literature, where early components (~150–250 ms) have been linked to early visual processing [41], and later components (~350 ms) to attentional and motivational salience processing in the insula [42,43]. This combined approach allowed us to identify plausible time windows while remaining sensitive to the specific dynamics of insular responses.

In this study, the mean amplitude method was employed for quantifying brain activity within the intervals. This technique was favoured over peak amplitude measurement due to its resistance to latency variability and high-frequency noise, as noted by Luck (2014) [40]. Subsequent statistical analyses were performed in SPSS 28.00. In the group analysis Section 2.4.1, a comprehensive dataset was compiled, encompassing mean amplitudes associated with every stimulus available for every bipolar pair of electrodes (N = 60), at both the early and late ERP intervals. This dataset was constructed using unsigned (absolute) amplitude values to assess signal magnitudes irrespective of their polarity. This methodology was selected at this stage in response to significant inter-individual and inter-electrode variability in insula activation, which posed a challenge to the homogeneity of variance assumption critical for ANOVA analyses. This approach aligned with our research question, which focused on comparing the magnitude of insular activity. Furthermore, the use of unsigned data counteracted the potential masking of activation patterns due to phase-cancellation effects. In the within-subject correlation analysis section (Section 2.4.2), the reduced variability permitted the use of signed amplitude values, enabling a more nuanced exploration of individual neuronal response patterns and correlations.

### 2.4. Statistical Analyses

#### 2.4.1. Group Analyses

In this section, a significance threshold of *p* ≤ 0.05 was employed, and all mean amplitudes were sourced from unsigned data.

The initial set of between-group analyses examined differences in insular responses to food versus non-food images across two prandial states (hungry and satiated). Paired-sample t-tests were used to compare the mean amplitudes of responses to food images against those to non-food images for both early and late ERP components. Given our directional hypothesis—that food images would elicit greater insular activation than non-food images—we employed a one-sided *t*-test. This was accompanied by a 90% confidence interval, providing a minimum estimate of the effect size at its lower bound.

Furthermore, we investigated variations in insular response to food images between prandial states. Paired-sample *t*-tests compared the mean amplitude of responses to food images between the hungry and satiated states for each ERP interval. Similarly to the previous t-test, given our directional hypothesis—that food images would generate a higher magnitude of insular activation in the hungry state compared to the satiated state—the t-tests conducted were one-sided. To identify specific insular regions contributing to these changes, a Multivariate Analysis of Variance (MANOVA) was conducted. This analysis used the mean amplitudes as dependent variables and the insular subregions (anterior, middle, and posterior) as independent variables. The choice of using a MANOVA was motivated in part by our hypothesis that insular subregions may exhibit distinct but potentially interrelated patterns of activation in response to food cues, and thus benefit from an analysis that can model these relationships jointly.

#### 2.4.2. Exploratory Correlation Analyses

In this section of the analysis, all mean amplitudes were sourced from signed data.

Exploratory correlation analyses were conducted to examine the relationship between insular responses to food images and the subjective ratings provided by participants. Given the exploratory nature of this section, the significance threshold was set at *p* ≤ 0.01, allowing for a liberal approach while still addressing the issue of multiple comparisons. Analyses were individualized for each participant, exploring correlations across each bipolar electrode pair, and across the four study conditions (hungry, satiated; early, late intervals). Partial correlations were employed, controlling for image characteristics (object size, brightness, contrast, complexity, and spatial frequencies).

The same statistical methodology was applied to explore the correlation between insular responses to food images and their objective nutritional values. Nutritional variables initially selected for analysis included the fat and carbohydrate content (per 100 g and in total), as well as the calorie content (in total). A preliminary correlation analysis was performed to ensure that all nutritional variables contributed uniquely to the analysis. Exploratory partial correlation analyses then assessed the relationship between these variables and brain activity, controlling for subjective ratings in addition to image characteristics.

To integrate these individual results, chi-square tests were used to compare the occurrence of significant correlations across all participants, measuring their association with prandial state, ERP interval, and insular anatomical location. For this part of the analysis, the significance threshold was set at *p* ≤ 0.05.

## 3. Results

### 3.1. Group Analyses

For the analysis, 76 contact points (forming 60 bipolar pairs) were retained, distributed across the anterior (14 right, 8 left), middle (9 right, 4 left), and posterior (16 right, 9 left) insular regions.

Table 2 presents the results of the one-tailed paired-sample t-tests comparing the mean amplitudes following the presentation of food versus non-food stimuli. In the hungry condition, significant differences were observed in the unsigned mean amplitudes for both the early and late ERP intervals. Food images elicited increased amplitudes compared to non-food images in both the early (90% CI lower bound = 0.11; Cohen’s d = 1.80) and late (90% CI lower bound = 0.31; Cohen’s d = 1.86) latency intervals. In the satiated condition, a significant difference was noted only in the early interval, with food images leading to increased amplitudes compared to non-food images (90% CI lower bound = 0.28; Cohen’s d = 1.85).

Table 3 displays the results from the one-tailed paired-sample t-tests examining the differences in the mean ERP amplitudes following the presentation of food according to the different prandial states. Significant differences were noted in the unsigned mean amplitudes across both the early and late intervals. Contrary to our predictions, the early interval revealed a lower unsigned mean amplitude in the hungry condition compared to the satiated condition (90% CI lower bound = −0.96; Cohen’s d = 2.08). This unexpected finding suggests a more complex interaction between the prandial state and neural response in the early post-stimulus interval than we initially hypothesized. In the late interval, consistent with our expectations, food images elicited higher amplitudes when participants were hungry compared to when they were satiated (90% CI lower bound = 0.06; Cohen’s d = 1.97).

Table 4 presents the MANOVA results comparing the mean amplitudes across anatomical locations under varying conditions (hungry vs. satiated; early vs. late intervals). Though there was no significant difference observed (*p* = 0.25) in the overall model, the mid-insula displayed the highest average of insular activation in all study conditions.

### 3.2. Exploratory Correlation Analyses

Table S1 presents the preliminary correlation analyses examining the relationship between the nutritional variables. A very strong correlation between the total fat content and total caloric content (*r* = 0.88) was noted. The total caloric content was deemed more informative as it provided information on the energy potential, regardless of the nutritional source. Thus, the variable for total fat content was not retained for further analyses.

Tables S2 and S3 present the exploratory partial correlation analyses, performed for each participant individually, examining the relationship between the insular activity of each pair of bipolar electrodes and the subjective self-report ratings, as well as the nutritional value. When controlling for the subjective ratings, 77% (17/22) of the significant correlations between electrode activity and nutritional value remained significant at the established *p* ≤ 0.01 threshold.

Table 5 presents the results of the crosstabulations performed to compare several of the study variables with the occurrence of significant correlations linking insular activity to subjective ratings. A significant association was observed between the occurrence of significant correlations and anatomical location: *X*^2^ (1, *N* = 480) = 6.90; *p* = 0.03; and Cramer’s V = 0.120. Brain activity correlating with hunger ratings appeared to be overrepresented in the posterior region (7/7), and that with palatability ratings in the mid-insula (3/4, 75%). It is also worth noting that significant correlations between insular activity and subjective reports occurred almost exclusively in the satiated condition (8/11, 73%) and in the right hemisphere (10/11, 91%), but these associations were not statistically significant (*p* = 0.13 and *p* = 0.11, respectively). There was no significant association between occurrences of significant correlations and a specific ERP interval.

The chi-square tabulations performed to compare the occurrences of significant correlations between insular activity and nutritional values (Table 6) revealed an association with the prandial state, *X*^2^ (1, *N* = 960) = 6.70, *p* = 0.01, and Phi = 0.08, with a higher representation of significant correlations in the hungry condition (17/22, 77%). There was no significant association with the laterality, ERP interval, or anatomical location. However, a difference according to the anatomical location was observed when comparing the occurrences of correlations for specific nutritional values rather than nutritional values as a whole category: *X*^2^ (6, *N* = 22) = 14.88; *p* = 0.02; and Cramer’s V = 0.58 (Table S4). The correlations between brain activity and carbohydrate content per 100 g appeared to be overrepresented in the posterior insula (4/4), and those with total calorie content in the mid-insula (3/4).

## 4. Discussion

In this exploratory study, we investigated the insula’s role in processing external food cues by analyzing the ERPs from intracranial EEG recordings in eight patients with epilepsy. We found that the prandial state significantly modulated insular responses to food images, with distinct effects observed in the early (150–250 ms) and late (350–450 ms) ERP intervals. Specifically, the response to food cues during hunger was greater than that during satiety in the late interval, whereas satiety was associated with greater responses in the early interval. Consistent with our predictions, insular activity related to the nutritional content of food cues was largely uncorrelated with the subjective value ratings, suggesting a separate neural encoding of implicit nutritional information and explicit subjective experiences. Additionally, the mid- and posterior insula exhibited stronger activation to both the subjective and nutritional properties of food cues compared to the anterior insula, which showed comparatively reduced activity. Together, these findings support the hypothesis that the insula plays a key role in the pre-ingestive evaluation of food, integrating learned post-ingestive outcomes, dynamically adjusting to homeostatic needs, and differentiating between nutritional and subjective value signals.

### 4.1. The Insula’s Response to External Food Cues Is Modulated by Changes in Homeostasis

Drawing on previous research showing increased insular activity in fasted individuals that decreased upon satiation [44,45,46,47], we expected pronounced responses in the hungry condition immediately after stimulus presentation (150–250 ms) and in the subsequent interval (350–450 ms). Contrary to our expectations, we observed a stronger insular response during satiation in the early interval, while the later phase was characterized by increased activation during hunger, as predicted.

The EPIC framework suggests that heightened insular activation in response to food-related stimuli during hunger may be a result of a prediction error, reflecting a significant mismatch between expected and actual physiological states [17]. Our findings of a stronger activation in the early interval (150–250 ms) when satiated could indicate a fast-responding inhibitory mechanism, whereby the insula recognizes a match between the current and ideal physiological states, potentially moderating the drive for food intake. This explanation is supported by research indicating that insular activity is associated with satiety-induced food aversion [45]. Conversely, in depressed individuals who exhibit increased appetite, low insular activity is associated with enhanced pleasure from food and an overactive reward system, suggesting that impaired insular functioning may allow an unchecked reward system to prevail [48]. In the same vein, a meta-analysis suggested that children’s diminished ability to resist tempting foods may be linked to their underactive insulae and overactive orbitofrontal cortices compared to adults [49]. It is worth noting that this early response may reflect a pure top-down modulation or attentional gating processes, rather than a prediction error per se—highlighting the need for further research to clarify the functional significance of early insular responses.

In the later phase (350–450 ms), the predicted increase in insular activation during hunger was indeed observed and may reflect the increased incentive value relative to the food cues, as well as attentional bias towards the stimuli (positive alliesthesia). In another study utilizing ERPs derived from iEEG recordings, this particular time window was associated with a p300-like response in the anterior insula in response to static sexual images, interpreted as increased attentional salience, and shown to contribute to the emergence of the late positive potential, which was thought to reflect facilitated attention to emotional stimuli [42,50]. Given that the bulk of evidence for intensified insular activity during energy deficits stems from fMRI studies—where BOLD signal enhancements typically peak several seconds post-stimulus—this increase in salience around the 400 ms mark could contribute to the subsequent amplification of neuronal activity within the insula.

Applying the predictive coding model and EPIC framework to our findings is challenging, primarily because the mechanisms of predictive coding and error signalling at the neuronal level remain largely undefined. In fMRI studies, interpreting BOLD signal increases as error signalling has been debated [51]. In EEG research, it has been suggested that the P3b component reflects prediction errors, following an earlier component influenced by stimulus salience [52]. The research on neuronal processes in the insula before 300 ms is limited, but a visual oddball task using iEEG revealed heightened insular activity in response to target stimuli within the 250–338 ms range [43]. This significant p300-like response, linked to salience detection and error monitoring [53], could represent a neuronal match between expected and detected inputs. Another intriguing research direction involves the study of pupil dilation under constant illumination, considered a measure of the noradrenergic system’s response to prediction errors [54,55,56,57]. Substantial evidence links insular activity to pupil dilation following prediction errors [56,58,59,60], a relationship that is supported by the connections between the locus coeruleus—the primary noradrenaline source—and the insula [61]. The possibility of a prediction signal emerging in the insula in the 150–250 ms range is consistent with the reported onset of pupil dilation, as early as 200 ms post-stimulus [56,62].

### 4.2. The Mid-Insula, a Food Cue Integration Hub?

We posited that the mid-insula would manifest the highest magnitude of activation in response to food stimuli when compared to other insular subregions, influenced by the body’s homeostatic state, while expecting the anterior insula to maintain steady activation levels regardless of such changes. Ultimately, we found no significant variations in activation by condition and anatomical location, possibly due to the small sample size. However, the mid-insula showed the highest average activation across all the conditions. Furthermore, the exploratory individual-level analyses revealed a connection between the activities of the mid- and posterior insula with the participants’ subjective experiences and the food’s nutritional content, a connection largely absent from the anterior insula.

These findings, albeit preliminary, align with theories proposing that the anterior insula specializes in gustatory function, representing taste intensity and quality independently from motivational and homeostatic modulation [8], or that it acts as a prediction generator that adjusts slowly to internal changes, receiving prediction errors submitted by the mid-insula [17]. Our results also underscore the dynamic response of the insula’s caudal regions, particularly in the mid-insula, corroborating studies that have highlighted its modulation by homeostatic signals [14,63] and its responsiveness to changes in a food’s reward value [45]. The mid-insula’s ability to integrate gustatory and interoceptive information within specialized multimodal neurons [15,16] and to respond readily to homeostatic changes has led researchers to posit that it serves as the integration site for general food perceptions—such as taste, sight, and smell—with the body’s internal state, thereby modulating gustatory cortex activity in an energy-conscious manner [64].

### 4.3. Tying Insular Activity to Subjective Experiences and Implicit Nutritional Data

In our investigation into the ties between insular activity and both the subjective assessments of and objective food characteristics, we discovered distinct connections: the posterior insula’s activity closely corresponded with hunger, while the mid-insula’s activity aligned with the subjective appeal of foods. Experiencing hunger at a conscious level likely involves an individual assessing their body’s state, gathering a variety of hunger cues from the stomach (e.g., growling, contractions) and mouth (e.g., saliva production), and the general emotional arousal (e.g., cravings) prompted by food [65,66,67]. These signals are communicated via interoceptive, somatosensory, and autonomic pathways, with the posterior insula acting as the primary receiver of such cues [68,69]. Hence, its connection to hunger evaluation is plausible, as it appears to integrate these interoceptive signals related to the physical sensations of hunger.

The appraisal of food’s palatability likely involves the brain’s valuation systems, influencing our “liking” and “wanting” responses (Morales & Berridge, 2020 [70]). The mid-insula’s role in regulating food intake by adjusting reward expectations has been highlighted by several fMRI studies, including Small et al.’s 2001 study [45], where it was found that the reward value of chocolate, as influenced by the mid-insula, diminished with satiation. Similarly, Simmons, Burrows, Avery, Kerr, Bodurka, Savage, and Drevets [48] observed that obese individuals with major depressive disorder rated food cues as more enjoyable, correlating with lower mid-insula activity and increased reward circuit activity, suggesting dysregulation when the mid-insula malfunctions. In the same vein, Dimitropoulos et al. [71] noted an absence of decreased mid-insular activation after eating in obese individuals, contrasting with healthy-weight counterparts. Furthermore, the case study of a patient with a stroke affecting the mid-to-posterior insula, who subsequently lost their appetite and developed anhedonia toward food [72], highlights the mid-insula’s importance in regulating food enjoyment and appetite. Similarly, the report of significant appetite loss post-insulectomy, where the maximal lesion overlap was in the mid-to-posterior insula [73], further supports this notion of a critical role in meal termination and food consumption regulation. Overall, these findings suggest that the mid-insula adjusts the perceived reward value of foods through its connections with the brain’s reward systems [67,74].

When examining the insula’s response to the nutritional content of food cues, we found that the anticipation of sugar, with its distinct and precise sensory experience of sweetness, appears to be tied to viscerosensory processing within the posterior insula, a finding that has been previously reported in gustatory stimulation fMRI paradigms [75,76,77]. On the other hand, the complex assessment of the total energetic content of food, which likely involves synthesizing the various indicators of an energy-rich meal, appears to necessitate the involvement of the mid-insula. This is in line with the mid-insula’s role in integrating a broad spectrum of food-related data, including primary sensory cues [10,13,16], homeostatic needs [14,78], and reward signals [45,48]. Unexpectedly, our findings show that the insula’s sensitivity to the nutritional value of food is more pronounced during hunger, highlighting a significant contrast to its activity during satiated states, when it relates more to subjective food evaluations. This suggests that hunger sharpens the brain’s focus on nutritional analysis, in line with prior studies that have linked increased cerebral investment in food assessment to hunger [79] and a stronger attentional bias towards food under similar conditions [80]. When hungry, afferent information from attentional, motivational, and homeostatic regions may upregulate insula activity, prioritizing the detection of the caloric and nutritional information crucial for the immediate physiological needs. Conversely, upon satiation, food valuation likely shifts from physiological necessities to factors like food’s utilitarian properties, personal preferences, past experiences, and cultural influences [81,82].

### 4.4. Nutritional Content Signalling Within the Insula Operates Largely Independently of Subjective Value

Finally, we found that the insula’s responsiveness to the nutritional value of food cues operates independently of subjective experiences. This finding supports the notion that nutrient sensing in the gut can enhance the reward value of foods beyond their hedonic qualities, without relying on subjective experience per se [27]. Studies on flavour–nutrient conditioning have shown that flavours associated with calories influence food intake more than taste alone [35], and that brain circuits respond to calorie-predictive flavours without altering the liking for them [83]. This aligns with findings that insulin release after eating, rather than taste hedonics, triggers the strongest reward responses in the striatum [84]. The discovery of neuropod cells in the gut and the pathways by which fat is sensed in the upper intestine have further underscored how visceral signals are transmitted to the brain, primarily through the vagus nerve, influencing behaviour through an interoceptive reward system [85,86]. Ultimately, these signals reach the insula, where they are integrated with other food-related stimuli.

### 4.5. Limits

Using absolute values to quantify EEG activity in our study facilitated inter-participant comparisons in the face of marked variability in insular response patterns. However, this approach did not capture polarity information, potentially obscuring more complex or directionally specific neural dynamics. Moreover, the small sample size (N = 8), while typical for intracranial EEG research, imposed significant limitations on the statistical power and generalizability. This is especially relevant for the subregion-level analyses (e.g., anterior vs. mid-insula), where the limited spatial sampling and inter-individual variability may have reduced our ability to detect subtle functional distinctions. The findings from these comparisons should thus be interpreted with caution. In addition, our participant population—epilepsy patients undergoing pre-surgical evaluation—introduced clinical variability that may have affected neural processing. Longstanding epilepsy and interictal activity may result in functional reorganization through neuroplasticity, especially in focal epilepsy [87,88]. The use of antiseizure medications may also have a significant impact on food cue reactivity in the insula, a variable that could not be controlled for due to the high variability in drug intake among the participants. Furthermore, the use of ad libitum breakfasts to produce the satiety condition may have introduced variability in the postprandial states. Ideally, future studies should consider using a standardized meal protocol when feasible, combined with physiological markers of satiety. Finally, the exploratory nature of this study and the multiple comparisons involved increased the risk of type I errors. Taken together, these factors necessitate caution in extrapolating our findings to a broader population, and underscore the need for future replication in larger, non-clinical samples.

## 5. Conclusions

To our knowledge, this study is the first to investigate the insula’s response to food cues using direct iEEG recordings. This exploratory study pushes our understanding of the insula’s multifaceted role in food perception, affirming its pivotal involvement in adapting to changes in the body’s homeostatic state, and in distinguishing between the nutritional content and subjective appeal of foods. Furthermore, our results reveal nuanced responses within the insula to external food cues, with different regions of the insula engaging distinctly in processing hunger signals and subjective experiences. Clinically, these findings highlight the need to look deeper into the potential of intervention-based approaches to modulate insular activity in individuals with disordered eating, such as real-time functional magnetic resonance imaging (rt-fMRI), neurofeedback training, and deep transcranial magnetic stimulation (dTMS), which have shown some promise thus far [89,90]. Indeed, although speculative, our findings raise the possibility that dTMS targeting the insula could influence eating behaviours by modulating early interoceptive or evaluative responses to food cues. For instance, dTMS over the insula and lateral prefrontal cortex has been shown to reduce cue-induced cigarette consumption [91], and preliminary studies on anorexia nervosa have suggested its potential for reducing compulsive food-related thoughts and improving mood and anxiety symptoms [90]. Whether therapeutic effects result from enhancing satiety signalling, dampening craving responses, or altering interoceptive precision remains an open question—but one that could be informed by further mapping the functional specificity of the insular subregions. Finally, an important future direction involves examining how stable psychological traits—such as impulsivity, cognitive flexibility, or interoceptive awareness—may modulate insular responses to food cues. These trait-level variables could help account for inter-individual variability in insular activity, particularly in how subjective and nutritional value signals are weighted. Incorporating such dimensions could contribute to a more embodied understanding of food valuation, bridging dynamic physiological states with more stable cognitive–emotional profiles.

## Figures and Tables

**Table 1:** Descriptive characteristics of the study participants (N = 8).

Pt.	Sex	Age (y)	Age 1st Sz (y)	Extra-Ins. Con.	Ins. Con.	Bipolar Con. for Analysis	Pre-Surgical Cerebral MRI Findings	Sz. Focus	Post-Testing Surgery
1	f	37	23	R (C, F); L (C, F)	27	14 (7 R [2 aI, 1 mI, 4 pI]; 7 L [1 aI, 1 mI, 5 pI])	Normal	R cingulate	R cingular cortectomy
2	m	54	22	R (F, T)	15	8 R (4 aI, 1 mI, 3 pI)	Normal	R hippocampo-amygdalar	None
3	f	33	2	L (F, T)	13	10 L (6 aI, 2 mI, 2 pI)	Normal (s/p L antero-temporal lobectomy 2 y prior)	L temporo-insular	L temporal lobectomy and L inferior insulectomy
4	m	30	24	R (F, T, P, O)	9	5 R (4 aI, 1 mI)	R occipital nodules	R occipital (nodules)	Laser ablation of R occipital nodules
5	m	36	14	R (F, C)	12	6 R (1 mI, 5 pI)	Normal	Unclear	None
6	m	57	25	R (T, O); L (T, O)	14	10 (6 R [5 aI, 1 mI]; 4 L [2 aI, 1 mI, 1 pi])	Encephalomalacia in bilateral occipital region	Hippocampi (bilateral)	None
7	m	44	12	R (F, T)	4	3 R (3 aI)	R cortical dysplasia type II	Unclear (possibly bitemporal)	R frontal cortectomy
8	f	21	14	R (C, F)	8	4 R (2 mI, 2 pI)	Normal (s/p R frontal cortectomy 3 y prior)	R frontal and R centro-temporal	Completion of R frontal cortectomy

Abbreviations: R, right; L, left; F, frontal; P, parietal; O, occipital; T, temporal; C, cingulate; aI, anterior insula; mI, mid-insula; pI, posterior insula; s/p, status post.

**Table 2:** Results of paired-sample t-tests comparing mean amplitudes of insular response to food and non-food stimuli (N = 60).

Prandial State	ERP Interval	Stimuli	Signed (+/−) Amplitude		Unsigned (+) Amplitude		Paired Differences (Unsigned Values)				
			M	SD	M	SD	M	SD	t	df	*p*
Hungry	150–250 ms	Food	0.56	2.33	9.87	3.80	0.50	1.80	2.15	59	0.018
		Non-Food	−0.14	3.77	9.37	3.51					
	350–450 ms	Food	0.61	4.43	10.95	4.21	0.71	1.86	2.97	59	0.002
		Non-Food	0.13	3.77	10.24	4.25					
Satiated	150–250 ms	Food	0.46	3.69	10.38	3.98	0.68	1.85	2.86	59	0.003
		Non-Food	−0.33	3.56	9.70	3.79					
	350–450 ms	Food	0.68	4.33	10.47	3.88	−0.17	2.47	−0.54	59	0.295
		Non-Food	0.19	4.31	10.64	4.38					

**Table 3:** Results of the paired-sample t-tests comparing the magnitude of the insular response according to the prandial state (N = 60).

ERP Interval	Prandial State	Signed (+/−) Amplitude		Unsigned (+) Amplitude		Paired Differences (Unsigned Values)				
		M	SD	M	SD	M	SD	t	df	*p*
150–250 ms	Hungry	0.56	2.33	9.87	3.80	−0.51	2.08	−1.90	59	0.031
	Satiated	0.46	3.69	10.38	3.98					
350–450 ms	Hungry	0.61	4.43	10.95	4.21	0.49	1.97	1.92	59	0.030
	Satiated	0.68	4.33	10.47	3.88					

**Table 4:** Comparison of amplitudes (M, SD) across anatomical locations and according to experimental conditions (*N* = 60).

ERP Interval	Prandial State	Anterior (*n* = 22)		Middle (*n* = 13)		Posterior (*n* = 25)		F (4, 55) ^a^
		Signed	Unsigned	Signed	Unsigned	Signed	Unsigned	
150–250 ms	Hungry	0.12 (2.37)	10.00 (4.59)	1.63 (2.92)	11.27 (2.32)	0.38 (1.84)	9.03 (3.55)	1.54
	Satiated	0.69 (4.24)	10.45 (4.95)	2.07 (4.54)	12.13 (2.97)	−0.57 (2.21)	9.42 (3.23)	2.06
350–450 ms	Hungry	0.64 (4.36)	11.10 (4.84)	2.77 (4.26)	12.12 (2.61)	−0.54 (4.32)	10.22 (4.28)	0.89
	Satiated	0.97 (4.88)	10.59 (4.89)	1.40 (4.19)	12.00 (2.40)	0.06 (3.98)	9.56 (3.34)	1.75

Note: ^a^ MANOVA performed on unsigned mean amplitude values.

**Table 5:** Crosstabulations examining the association between the study variables and the occurrence of significant correlations relating brain activity to subjective ratings (hunger and palatability ratings on 5-point bipolar Likert scales).

		Significant Correlation		Crosstabulation		
Variables		Yes	No	df	X^2^	*p*
Prandial state	Hungry	3	237	1	2.33	0.13
	Satiated	8	232			
ERP interval	Early	6	234	1	0.09	0.76
	Late	5	235			
Laterality	Right	10	302	-	- ^a^	0.11
	Left	1	167			
Anatomical subregion	Anterior	0	176	-	8.07 ^b^	0.01
	Middle	3	101			
	Posterior	8	192			

Note: ^a^ Fisher’s exact test; ^b^ Fisher–Freeman–Halton exact test.

**Table 6:** Crosstabulations examining the association between several study variables and the occurrence of significant correlations relating brain activity to nutritional value (fat/100 g, carbs/100 g, fat total, carb total, calorie total).

		Significant Correlation		Crosstabulation		
Variables		Yes	No	df	*X* ^2^	*p*
Prandial state	Hungry	17	463	1	6.70	0.01
	Satiated	5	475			
ERP intervals ^a^	Early	8	472	1	1.68	0.20
	Late	14	466			
Laterality	Right	15	609	1	0.1	0.75
	Left	7	329			
Anatomical subregion	Anterior	6	346	2	0.95	0.62
	Middle	6	202			
	Posterior	10	390			

Note: ^a^ Early interval: 150–250 ms, Late interval: 350–450 ms.

## Data Availability

The data presented in this study are available on request from the corresponding author, as they originate from clinical recordings obtained during the pre-surgical evaluation of patients with epilepsy.

## Supplementary Materials

The following supporting information can be downloaded at: https://www.mdpi.com/article/10.3390/brainsci15060593/s1, Table S1: Preliminary correlation analysis examining the correlation between the selected variables; Table S2: Partial correlations examining the relationship between insular activity and nutritional value; Table S3: Partial correlations examining the relationship between insular activity and subjective ratings; Table S4: Crosstabulations examining the association between specific nutritional content and the occurrence of significant correlations relating brain activity to nutritional value.
