# Supplementary material for: Separating Subjective from Objective Food Value in the Human Insula: An Exploratory Study Using Intracranial EEG
Source: Brain Sci. 2025 May 31;15(6):593. doi: 10.3390/brainsci15060593 (PMC12191078; doi:10.3390/brainsci15060593)
Supplement: Supplementary file 1 [file brainsci-15-00593-s001.zip › brainsci-3645520-supplementary.pdf]

Table S1. Correlation analysis examining the correlation between selected variables.

Variable	n	1	2	3	4	5	6
1. Fat per 100g	141	1					
2. Carbs per 100g	141	.36*	1				
3. Kcal per 100g	141	.86*	.76*	1			
4. Fat total	140	.57*	-.02	.38*	1		
5. Carbs total	139	.09	.35*	.23*	.37*	1	
6. Kcal total	137	.42*	.07	.36*	.88*	.57*	1

*p < .01.

Table S2. Partial correlations examining the relationship between insular activity and nutritional value

			Hungry Condition								Satiated Condition							
			Fat/100g		Carbs/100g		Carbs total		Kcal total		Fat/100g		Carbs/100g		Carbs total		Kcal total	
**Electrode**	**Hem**	**Area**	**Early**	**Late**	**Early**	**Late**	**Early**	**Late**	**Early**	**Late**	**Early**	**Late**	**Early**	**Late**	**Early**	**Late**	**Early**	**Late**
P1 31-32	L	A	.27	.36	-.06	-.02	.15	.09	.21	.30	-.07	.30	.20	.00	.25	-.27	-.35	-.03
P3 12-13	L	A	-.04	-.13	.03	.24	.23	**.59*†**	.30	.40	.12	-.02	.08	-.11	.05	-.10	.06	-.10
P3 13-14	L	A	-.14	-.07	.05	.27	.14	**.63*†**	.15	.31	.18	.03	.06	.11	-.08	.01	-.10	-.03
P3 14-15	L	A	-.40	-.09	-.04	.19	-.08	.00	-.10	-.11	-.36	-.14	-.24	-.02	-.03	.13	-.19	.02
P3 15-16	L	A	.04	-.21	-.28	-.34	-.27	-.35	-.20	-.27	**.55***	.16	.15	-.02	.13	.22	.36	.29
P3 31-32	L	A	-.22	-.13	-.20	.12	-.13	.13	.02	.04	.12	.10	.03	-.10	.16	.02	.17	.16
P3 32-33	L	A	.03	-.33	-.36	-.14	-.12	-.14	-.01	-.29	.15	.01	.19	.16	.22	.23	.35	.15
P6 11-12	L	A	.08	-.32	-.07	-.03	-.21	-.03	-.25	-.13	-.06	-.15	.26	.24	-.03	.05	.17	.17
P1 23-24	R	A	.29	.23	-.01	.15	-.11	-.11	.06	.01	.07	.31	.22	.37	.37	.33	.18	.33
P1 24-25	R	A	-.11	-.31	.15	-.03	.02	.17	.08	.21	.05	-.01	.07	.08	-.01	**-.62***	.09	.03
P2 41-42	R	A	.32	.23	.11	.06	-.06	.05	.09	-.04	-.23	-.13	.09	.29	.10	.10	-.30	-.30
P2 42-43	R	A	.27	**.39*†**	.03	.14	-.03	.05	.17	.02	-.06	-.05	.24	.25	.16	.02	-.05	-.19
P2 43-44	R	A	.14	.16	.10	.07	-.03	-.03	-.08	-.13	.14	.01	.11	.22	.06	-.09	.15	-.17
P2 44-45	R	A	-.09	.01	-.06	-.08	-.17	-.16	-.09	.14	-.08	-.11	-.02	-.21	.03	.12	.04	.07
P4 21-22	R	A	.15	-.09	-.24	-.23	.32	.18	.38	.20	.10	-.23	.01	-.17	-.25	-.25	-.10	-.27
P4 22-23	R	A	-.32	.08	.07	.09	.06	.00	-.02	-.01	-.03	.04	.27	.31	.33	.09	.22	.00
P4 23-24	R	A	-.13	.19	.28	.17	.15	.13	.10	.16	-.02	-.22	.11	-.21	.00	-.17	.03	-.12
P4 24-25	R	A	.19	.21	.37	.09	.13	.01	.10	.04	-.18	-.02	.28	-.02	.26	.20	-.07	.13
P6 21-22	R	A	.24	-.36	-.22	-.18	.14	.14	.18	.16	-.04	.11	.05	.06	.11	-.09	.03	-.10
P7 22-23	R	A	-.20	.05	.09	.23	-.05	.12	-.23	.01	.07	.23	.01	.07	-.11	-.06	-.03	-.04
P7 23-24	R	A	.05	.05	.03	-.04	-.01	-.10	-.11	-.17	-.05	-.05	.02	.02	-.07	-.07	-.16	-.16
P7 24-25	R	A	.00	-.08	-.27	-.09	**-.38***	-.31	-.20	-.17	-.11	-.26	.05	-.11	.03	.14	.08	.15
P1 32-33	L	C	.40	.10	.01	.03	.30	.20	.41	.27	-.04	.48	.23	-.08	.02	-.19	-.24	.30
P3 33-34	L	C	-.08	.28	-.31	-.12	.11	.43	.18	**.55*†**	-.11	.05	-.33	-.17	-.16	.05	-.25	.01
P3 34-35	L	C	.14	.20	.21	.20	.02	.12	-.05	.05	-.36	.09	-.01	.33	.13	-.08	.02	-.19
P6 12-13	L	C	**.62*†**	.30	.01	.18	.06	-.05	.21	-.23	.27	.27	-.31	-.18	-.21	-.30	-.03	-.06
P1 41-42	R	C	-.08	.13	-.14	.00	.06	.11	.12	.08	.05	-.14	-.25	.13	-.14	.36	-.38	-.15
P2 61-62	R	C	.00	.04	.12	-.01	.04	-.21	-.02	-.12	-.03	-.13	.07	-.09	.00	-.15	.25	.05
P4 41-42	R	C	**-.42*†**	-.04	.12	.15	.08	-.06	.01	-.07	.04	.15	.12	.23	.28	.13	.28	.09
P5 41-42	R	C	-.13	-.24	-.20	-.08	-.18	-.21	-.16	-.21	.09	.07	.36	.04	-.15	-.29	-.18	-.18
P6 22-23	R	C	-.26	**-.51*†**	.29	.32	.13	.13	.01	-.08	-.21	-.02	-.11	-.07	-.08	.09	-.27	.11
P6 23-24	R	C	-.13	-.48	.31	.23	.25	.13	.34	-.02	-.25	.01	-.07	.00	.08	.06	**-.44*†**	.07
P6 24-25	R	C	.11	-.18	.32	.41	-.16	-.15	-.33	-.50	.20	.17	.21	-.15	.12	.03	.16	.32

P8 41-42	R	C	-.03	.30	.12	.14	.25	.07	.22	.21	.11	.08	-.20	.04	-.28	.09	-.08	.10
P8 42-43	R	C	.20	.14	.11	.24	.21	.00	.17	-.01	-.07	.07	-.21	.00	-.09	.03	.14	**.45***
P1 33-34	L	P	.14	.04	.17	.09	-.06	.02	.08	.01	.34	-.31	.37	.25	.02	.40	.27	.14
P1 34-35	L	P	.06	-.18	-.18	.13	-.23	-.20	.08	-.28	.09	-.10	.16	.13	-.32	-.35	.01	.38
P1 35-36	L	P	-.11	-.38	-.09	-.15	-.23	-.25	-.08	-.34	.09	.10	-.20	-.33	-.25	-.58	-.28	.05
P1 36-37	L	P	-.19	.03	-.03	-.09	.09	.01	-.02	.03	.10	.05	-.47	-.16	-.14	-.23	.23	-.14
P1 37-38	L	P	-.03	.09	-.14	-.06	.01	.09	.17	.14	-.46	-.29	.03	-.20	.01	.41	-.16	-.31
P3 35-36	L	P	-.11	.07	.21	.07	.21	.26	.04	.18	-.15	-.03	.02	-.13	-.01	-.44	-.20	-.42
P3 36-37	L	P	-.21	.06	.02	.13	-.22	.02	-.21	-.14	.19	-.09	**.57*†**	.04	.47	.08	.24	-.02
P6 15-16	L	P	-.41	-.01	-.27	-.41	-.09	.09	-.22	.17	-.18	-.09	-.36	-.04	.05	.13	.07	.09
P6 16-17	L	P	.37	.41	-.14	-.24	-.32	**-.52*†**	-.15	-.31	-.17	-.23	-.03	.02	-.13	-.34	-.22	-.23
P1 42-43	R	P	**-.43*†**	-.31	-.38	**-.44*†**	.02	.15	.11	.08	.01	.13	.11	-.10	-.03	-.14	-.10	.16
P1 43-44	R	P	.18	.13	.15	.02	-.12	-.29	-.27	-.26	-.04	-.18	.13	-.30	-.06	.08	.09	-.18
P1 44-45	R	P	.21	-.03	.20	.09	-.11	-.35	-.10	-.36	.18	-.21	-.28	.12	.02	-.09	.31	.06
P1 45-46	R	P	-.02	-.21	.28	.12	.39	.07	.12	-.01	.13	-.09	-.19	.20	.17	.00	.46	.02
P2 65-66	R	P	.05	.08	.12	.23	.11	.13	.17	.02	.09	.02	-.02	-.02	.05	.08	-.09	-.12
P2 66-67	R	P	.16	.20	.17	.13	.26	.10	.03	.04	-.09	-.14	.01	.07	.05	-.06	-.17	-.28
P2 67-68	R	P	.01	-.20	-.03	-.19	.15	-.02	-.02	-.05	-.03	.02	-.08	.07	.10	.03	.14	.02
P5 42-43	R	P	**.50*†**	.12	.27	**.45*†**	.03	**.39***	.15	.20	-.08	-.25	.04	.11	-.06	.04	-.04	-.12
P5 43-44	R	P	-.39	-.17	-.36	-.34	-.28	-.09	-.26	.00	-.12	-.07	-.20	-.05	-.26	.03	-.16	.01
P5 44-45	R	P	-.36	-.04	-.35	-.19	-.14	-.25	-.13	-.21	.15	.14	-.03	.27	.13	.32	.28	.11
P5 45-46	R	P	-.01	-.15	.06	-.25	-.09	**-.55*†**	-.04	**-.52*†**	.14	.05	-.19	.04	.15	.12	.14	.01
P5 46-47	R	P	.38	.22	**.46*†**	.15	.16	-.32	.12	-.25	-.19	-.12	-.01	.09	.09	.32	-.32	.06
P6 25-26	R	P	-.37	-.26	-.04	-.20	.19	.09	.17	.21	-.26	-.19	-.26	-.21	-.19	-.14	-.37	-.30
P6 26-27	R	P	-.18	.14	.28	.10	.07	.03	.18	.08	-.22	-.24	-.03	.02	-.06	-.04	-.17	-.05
P8 43-44	R	P	-.09	.11	.01	.28	.01	-.03	.00	-.10	.25	.39	-.02	.15	.13	.14	.19	.01
P8 44-45	R	P	.03	.19	-.06	.09	.10	.09	.04	.18	-.04	.20	-.03	.19	.15	.00	-.05	.09

Note: *p < .01. †Remains significant (p < .01) after controlling for subjective (appetizing, hunger) ratings. Image characteristics (object size, brightness, contrast, complexity, spatial frequencies) were used as control variables.

Table S3. Partial correlations examining the relationship between insular activity and subjective ratings

			Hungry condition				Satiated condition			
			Palatability		Hunger		Palatability		Hunger	
**Electrode**	**Hem**	**Area**	**Early**	**Late**	**Early**	**Late**	**Early**	**Late**	**Early**	**Late**
P1 31-32	L	A	-0.08	0.10	0.04	0.06	-0.29	0.06	-0.37	-0.05
P3 12-13	L	A	0.00	0.02	0.29	0.26	-0.33	-0.31	-0.06	-0.07
P3 13-14	L	A	0.00	-0.07	0.06	0.12	-0.19	-0.22	0.01	-0.19
P3 14-15	L	A	0.09	-0.05	-0.03	-0.25	0.13	-0.07	0.12	-0.17
P3 15-16	L	A	-0.05	0.07	0.14	0.12	-0.32	-0.02	-0.12	0.28
P3 31-32	L	A	-0.06	-0.17	-0.01	-0.15	0.13	0.10	0.27	0.17
P3 32-33	L	A	-0.01	0.08	-0.17	-0.12	-0.38	-0.05	-0.19	-0.02
P6 11-12	L	A	0.01	0.06	-0.15	-0.02	0.12	0.10	0.11	0.14
P1 23-24	R	A	0.02	-0.02	0.15	0.02	-0.10	-0.14	-0.08	-0.25
P1 24-25	R	A	-0.07	0.05	0.00	0.05	0.02	0.13	-0.12	0.26
P2 41-42	R	A	0.09	-0.01	0.07	-0.05	-0.05	-0.03	-0.01	0.07
P2 42-43	R	A	-0.12	-0.10	-0.10	-0.10	-0.02	-0.06	0.02	-0.03
P2 43-44	R	A	-0.02	-0.12	0.06	-0.15	0.08	-0.08	0.08	-0.05
P2 44-45	R	A	-0.04	0.06	-0.06	0.07	0.10	0.05	0.09	-0.02
P4 21-22	R	A	0.06	0.00	-0.07	0.12	0.07	0.05	-0.12	-0.10
P4 22-23	R	A	-0.06	-0.06	-0.11	-0.20	-0.07	-0.21	-0.28	-0.26
P4 23-24	R	A	-0.07	0.04	-0.11	-0.21	-0.16	0.04	-0.31	-0.27
P4 24-25	R	A	-0.02	-0.03	-0.01	-0.13	-0.09	0.16	-0.29	-0.11
P6 21-22	R	A	-0.21	-0.04	-0.02	0.20	0.20	0.15	-0.05	-0.05
P7 22-23	R	A	-0.15	0.00	-0.09	-0.09	-0.03	0.17	-0.03	-0.15
P7 23-24	R	A	-0.08	-0.11	-0.07	-0.22	0.08	0.08	0.00	0.00
P7 24-25	R	A	-0.22	-0.10	-0.19	-0.12	0.00	-0.03	0.12	-0.04
P1 32-33	L	C	0.09	0.10	0.19	0.15	-0.23	0.05	-0.16	-0.02
P3 33-34	L	C	-0.08	-0.24	-0.22	-0.31	-0.18	0.09	0.16	0.46
P3 34-35	L	C	0.18	0.29	0.25	0.13	0.27	0.34	0.00	-0.01
P6 12-13	L	C	0.03	-0.09	-0.15	-0.27	-0.11	0.00	-0.14	-0.05
P1 41-42	R	C	-0.03	-0.21	-0.04	-0.23	-0.46	-0.29	-0.37	-0.20
P2 61-62	R	C	0.10	-0.06	0.01	-0.15	-0.32	**-0.41***	-0.23	-0.28
P4 41-42	R	C	0.01	-0.11	0.08	-0.13	0.06	-0.10	-0.16	-0.21

P5 41-42	R	C	-0.06	0.10	-0.15	-0.06	0.14	-0.01	0.00	-0.20
P6 22-23	R	C	-0.34	-0.18	0.04	0.07	0.08	0.06	-0.12	0.01
P6 23-24	R	C	-0.30	-0.21	-0.04	-0.01	-0.07	-0.02	-0.19	-0.20
P6 24-25	R	C	-0.25	-0.03	0.07	-0.07	0.02	-0.10	-0.18	-0.21
P8 41-42	R	C	-0.22	**0.50***	-0.34	0.25	0.25	0.16	-0.13	0.00
P8 42-43	R	C	0.19	**0.51***	-0.12	0.13	0.08	-0.20	0.16	0.04
P1 33-34	L	P	-0.09	0.10	-0.11	0.11	-0.12	-0.19	-0.14	-0.18
P1 34-35	L	P	-0.04	0.03	-0.06	0.02	0.12	0.13	0.07	0.18
P1 35-36	L	P	0.12	0.06	0.06	0.02	0.01	0.05	-0.08	-0.08
P1 36-37	L	P	0.16	-0.06	-0.05	-0.09	0.04	0.00	-0.12	0.05
P1 37-38	L	P	0.08	-0.22	0.05	-0.21	-0.05	-0.26	0.03	-0.22
P3 35-36	L	P	0.13	-0.06	0.15	-0.02	0.06	-0.19	-0.14	-0.51
P3 36-37	L	P	-0.23	-0.01	-0.19	0.02	0.11	-0.03	-0.03	-0.19
P6 15-16	L	P	-0.08	-0.23	0.04	-0.09	0.19	0.15	**0.36***	0.34
P6 16-17	L	P	-0.11	-0.06	0.07	-0.10	-0.14	-0.13	-0.04	-0.02
P1 42-43	R	P	0.05	0.06	0.12	-0.01	-0.26	-0.22	-0.23	-0.16
P1 43-44	R	P	-0.14	-0.12	-0.25	-0.11	0.35	0.12	0.23	0.02
P1 44-45	R	P	-0.05	-0.07	-0.03	0.04	0.45	0.38	**0.55***	**0.49***
P1 45-46	R	P	-0.20	0.01	-0.15	0.08	**0.49***	0.28	**0.53***	0.27
P2 65-66	R	P	0.30	0.16	0.22	0.16	-0.02	0.03	0.00	0.09
P2 66-67	R	P	0.00	-0.09	0.10	0.04	-0.21	-0.28	-0.15	-0.20
P2 67-68	R	P	0.01	-0.04	0.10	0.10	-0.27	-0.18	-0.23	-0.13
P5 42-43	R	P	-0.31	-0.07	**-0.37***	-0.11	-0.03	-0.21	-0.12	-0.26
P5 43-44	R	P	-0.06	0.02	-0.01	-0.04	0.16	0.21	0.21	0.33
P5 44-45	R	P	0.03	-0.12	0.01	0.00	-0.28	-0.07	-0.06	0.09
P5 45-46	R	P	0.03	0.05	0.15	0.10	0.12	-0.07	0.09	-0.03
P5 46-47	R	P	-0.17	0.01	-0.02	-0.04	-0.07	-0.09	0.10	-0.10
P6 25-26	R	P	-0.01	-0.08	-0.20	0.04	-0.01	-0.04	-0.08	0.02
P6 26-27	R	P	0.10	0.03	0.15	-0.15	-0.02	-0.06	0.04	0.03
P8 43-44	R	P	0.01	0.33	-0.14	0.15	0.17	0.10	-0.13	-0.03
P8 44-45	R	P	0.11	-0.14	0.23	0.12	0.29	0.04	**0.39***	**0.40***

*p < .01. Image characteristics (object size, brightness, contrast, complexity, spatial frequencies) were used as control variables.

Table S4. Crosstabulations examining the association between specific nutritional content and the occurrence of significant correlations relating brain activity to nutritional value

		**Significant correlation**				**Crosstabulation**		
		**Fat/100g**	**Carb/100g**	**Carb T**	**Kcal T**	**df**	**x2**	**p**
Anatomical subregion	Anterior	2	0	4	0	-	11.83	0.03
	Middle	3	0	0	3			
	Posterior	2	4	3	1			
